# Post-traumatic stress disorder, depressive symptoms, and cognitive function among middle-aged urban adults: Healthy Aging in Neighborhoods of Diversity across the Life Span study

**DOI:** 10.1007/s11357-025-01825-0

**Published:** 2025-09-15

**Authors:** Michael F. Georgescu, May A. Beydoun, Robert H. Pietrzak, Hind A. Beydoun, Nicole Noren Hooten, Sri Banerjee, Michele K. Evans, Alan B. Zonderman

**Affiliations:** 1 Laboratory of Epidemiology and Population Sciences, National Institute On Aging, Intramural Research Program, NIA/NIH/IRP, 251 Bayview Blvd., Suite 100, 04B118, Baltimore, MD 21224 USA; 2 Department of Psychiatry, Yale School of Medicine, New Haven, CT USA; 3 U.S. Department of Veterans Affairs National Center for Posttraumatic Stress Disorder, VA Connecticut Healthcare System, West Haven, CT USA; 4 Department of Social and Behavioral Sciences, Yale School of Public Health, New Haven, CT USA; 5 U.S. Department of Veterans Affairs, VA National Center On Homelessness Among Veterans, Washington, DC USA; 6 Department of Management, Policy, and Community Health, School of Public Health, University of Texas Health Science Center at Houston, Houston, TX USA; 7 Public Health Doctoral Programs, Walden University, Minneapolis, MN USA

**Keywords:** Post-traumatic stress disorder, Depressive symptoms, Cognitive function, Cross-sectional study, Older adults

## Abstract

Post-traumatic stress disorder (PTSD) and depressive symptoms Have been linked to cognitive functioning. However, the impact of PTSD and depression on cognitive functioning, especially among middle-aged urban adults, is not well understood. Cross-sectional data were analyzed from 1434 participants from the Healthy Aging in Neighborhoods of Diversity across the Life Span study. We used multivariable adjusted linear regression and structural equation models (SM) to test the association between PTSD, CES-D, and cognitive test scores. We found PTSD to be strongly associated with depressive symptoms despite adjustment for potential confounders. SM with cognitive test scores as final outcomes found Delayed Free Recall and List A immediate recall inversely associated with PTSD score, with TEs of *β* ± SE − 0.08398 ± 0.02654, *P* = 0.002, and *β* ± SE − 0.10734 ± 0.02571, *P* < 0.001, respectively, and IEs of *β* ± SE − 0.06786 ± 0.02202, *P* = 0.002, and *β* ± SE − 0.07625 ± 0.02140, *P* < 0.001, indicating that depressive symptoms significantly mediated the potential association of PTSD with poorer verbal memory. Other cognitive test scores showed significant total or direct effects (*p* < 0.05), suggesting different pathways not including depressive symptoms as a mediator. At higher intima-media thickness (IMT) tertiles, PTSD score was strongly associated with total CES-D score. This study provides evidence of an association between PTSD and cognitive functioning among middle-aged urban adults, partially mediated through depressive symptoms in verbal memory, with IMT enhancing the association between PTSD and depressive symptoms.

**Supplementary Information:**

The online version contains supplementary material available at 10.1007/s11357-025-01825-0.

## Introduction

Post-traumatic stress disorder (PTSD) Has historically been of public health interest. In the USA alone, over 80% of the population has been exposed to some traumatic event in their life [1], and over 8% reach the threshold of developing PTSD [2]. Individuals who develop PTSD, which is defined as a consequence of exposure to a traumatic event physically and/or psychologically that threatens one’s life [3–5], are often associated with poor concentration, hypervigilance, recurrent memories, flashbacks or nightmares, and being emotionally withdrawn [5]. The associations between PTSD and cognitive functioning have been well documented [6–8]. A recent systematic review reported mounting evidence of PTSD being a risk factor for dementia and imaging findings among those with PTSD-related dementia overlapping with Alzheimer’s disease patterns [9]. However, PTSD is clinically a misdiagnosed condition due to the commonly coexisting mental health condition of depression.

Depressive symptom severity has been linked to poor cognitive functioning [10–13], and previous studies have linked depression with increased risk of cognitive decline and dementia [13]. In a systematic review and meta-analysis among those with diabetes, depression was associated with poor cognitive functioning and increased risk of dementia [14]. Another systematic review and meta-analysis reported that both clinical and subthreshold depressive symptoms were related to poor cognitive function, especially among older ages [15]. Depressive symptoms have also been connected to PTSD in older adults in several studies [15–17] and were associated with greater intima-media thickness (IMT) in previous studies [18–21]. Carotid IMT is an indicator of subclinical atherosclerosis; therefore, given that studies have shown a significant association between subclinical atherosclerosis and depression or chronic stress [22, 23], measuring IMT and its connections to mood and stress-related symptoms is of clinical relevance.

Given the interdependence of PTSD, depressive symptoms, and cognitive function, the mediating paths between them, as well as potential interactions with health-related factors, become important research questions. Nonetheless, few studies have examined the mediating effect of depressive symptoms in the association between PTSD and cognitive function in an urban cohort of African American and White middle-aged adults. The present study investigated the cross-sectional associations of PTSD, depressive symptoms (total score and components), and cognitive function among urban middle-aged adults participating in the Healthy Aging in Neighborhoods of Diversity Across the Life Span study (HANDLS), while accounting for potential confounding factors (such as sociodemographic (e.g., age, sex, race/ethnicity), lifestyle (e.g., smoking status, drug use), and health related factors (e.g., body mass index (BMI) and comorbidities such as diabetes and myocardial infarction)).

We used multivariable-adjusted regression models to test three different pathways: PTSD to depressive symptoms, PTSD to cognition, and depressive symptoms to cognition. Importantly, we used structural equation models to test the mediating pathway between PTSD and 13 cognitive test scores through depressive symptoms, overall and across IMT tertiles, while accounting for potential confounding factors that may influence the associations (such as cardiovascular conditions: myocardial infarction, angina, coronary artery disease, atrial fibrillation, and congestive heart failure).

## Material and methods

### Database

The HANDLS study was initiated in 2004 by the Intramural Research Program (IRP) of the National Institute on Aging (NIA). The study is currently in progress and is a prospective cohort study aimed at examining health disparities in aging and age-related conditions. HANDLS is an interdisciplinary research study that examines a variety of domains in African American and White individuals living above and below poverty. It uses mobile medical research vehicles (MVRs) and state-of-the-art research devices to improve retention and engagement among non-traditional research participants. The HANDLS project was approved by the Institutional Review Board of the National Institutes of Health, and research participants provided written informed consent [24–32].

Between 2004 and 2009, baseline HANDLS data were collected in two waves (Visit 1). During the initial phase, the participants completed questionnaires and in-home interviews on their health, usage of health services, psychosocial variables, diet, characteristics of their area, and demographics. The second phase involved measuring laboratory parameters (blood chemistries, hematology, biomarkers of oxidative stress, biomaterials for genetic studies), psychophysiological assessments (heart rate variability, arterial thickness, carotid ultrasonography, assessments of muscle strength, bone density), medical history, physical examination, dietary recall, and cognitive evaluation. All of these procedures were performed in MRVs. After that, HANDLS participants were seen every 5 years; visit 2 took place between 2009 and 2013. HANDLS data segments may be found at https://handls.nih.gov/06Coll-w00dataDocR.cgi for analysis.

### Study measures

#### Cognitive function

At visit 1 of HANDLS, a variety of cognitive tests were employed to evaluate cognition, including the Mini-Mental State Examination (MMSE), the California Verbal Learning Test (CVLT) Immediate (List A) and Delayed Free Recall (DFR), the Benton Visual Retention Test (BVRT, # of errors), the Brief Test of Attention (BTA), the Animal Fluency test (AF), the Digit Span Forward and Backwards tests (DS-F and DS-B), the Clock Drawing Test (CDT), and the Trail Making Test Parts A and B (TRAILS A and B, in seconds), Card Rotation Test (CRT), and the Identical Pictures (IDP). Each cognitive assessment is thoroughly explained on GitHub at https://github.com/baydounm/COGNITIVE_TESTS_HANDLS and in previous studies (e.g., [33, 34]). The cognitive domains that were addressed were global mental state, verbal memory, verbal fluency, attention, visual memory, visuospatial ability, and executive function, which included working memory. The whole MMSE was normalized using the methods that were previously discussed [35]. The TRAILS A and B scores (in seconds) were Ln converted to achieve pseudo-normality. With the exception of the BVRT, TRAILS A and B, all cognitive test results pointed towards higher values, suggesting greater performance.

#### PTSD

At visit 1 of HANDLS, an audio computer-assisted self-interviewing (ACASI) PTSD checklist scale was administered. Participants were asked 18 psychometrically validated questions on PTSD symptoms and asked how strongly they endorse each question from “Not at all” to “Extremely.” As the main exposure variable of interest, we identified PTSD severity from the tertiles using the STATA command *xtile*; additionally, we used the STATA plugin (*mi estimate*) for each tertile of PTSD.

#### Depressive symptoms

Depressive symptoms were measured using the 20-item Center for Epidemiologic Studies-Depression scale (CES-D), which is described in Supplemental Material 2. The CES-D assesses four sub-scales of depressive symptoms that are affective, interpersonal problems, somatic complaints, and positive affect. We used the STATA plugin (mi estimate) developed from a well-established SAS approach [36] for total depressive symptoms and each sub-scale of depressive symptoms.

#### Intima-media thickness

A high-resolution B-mode ultrasonography for examination of the left carotid artery was completed to measure IMT among participants in cm. A high-resolution B-mode ultrasonography is a noninvasive, in vivo examination of the structure and function of carotid arteries. Additionally, if the study doctor discovered any condition or problem, the information was provided to the participants immediately and their primary care physician, with their permission. If the participants did not have a primary care physician, efforts would be made to refer them for care. Participants were reimbursed for their time.

#### Covariates

The Healthy Eating Index for 2010 (HEI-2010) and health (body mass index (BMI) weight/height^2^ in kg.m^−2^, continuous), comorbidities, allostatic load, and self-rated health were included when analyzing the proposed association between PTSD, depressive symptoms, and cognitive performance. Confounding factors included age, race (White, African American), poverty status (less than 125%, more than 125%, ≥ 125%) [37], education (less than high school, high school, more than high school), literacy (Wide Range Achievement Test, third edition (WRAT-3) (Supplemental Material 1), lifestyle (current cigarette smoking, yes, no), current drug use, yes, no (using any of the opiates, cocaine, and marijuana)), and the 2010 Healthy Eating Index (HEI-2010). Age was examined as a continuous variable. Poverty status was operationalized (37) using the Department of Health and Human Services poverty thresholds based on household income and total household size. The HEI-2010 (38) uses food and macronutrient-related factors to evaluate the overall quality of an American’s diet. Energy intake was assessed through a 2-day recall and then combined to obtain the mean energy intake of each individual. Comorbidities included self-reported history of any number of cardiovascular conditions, such as myocardial infarction, angina, coronary artery disease, atrial fibrillation, and congestive heart failure (0 = no, 1 = yes). Comorbidities were further defined as follows: diabetes (0 = non-diabetic, 1 = pre-diabetic, and 2 = diabetic), dyslipidemia (or statin usage; 0 = no, 1 = yes), and dyslipidemia. Classification for self-rated health was 0 for poor/average, 1 for good, and 2 for very good/excellent.

#### Sample

**Fig. 1:**
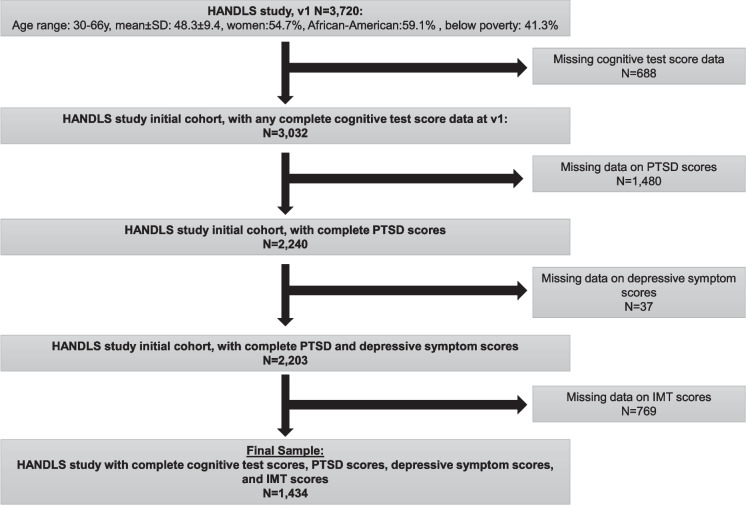
Participant flowchart. Abbreviations: HANDLS, Healthy Aging in Neighborhoods of Diversity across the Life Span; IMT, intima-media thickness; PTSD, post-traumatic stress disorder; v1, visit 1

As depicted in Fig. 1, HANDLS recruited an initial sample of 3720 participants (age range, 30–66 years; mean ± SD, 48.3 ± 9.4; women, 54.7%; African American, 59.1%; below poverty, 41.3%) at visit 1. We restricted the sample to include those with complete cognitive test scores (*N* = 3032), PTSD scores (*N* = 2240), depressive symptom scores (*N* = 2203), and IMT scores, leading to a final sample of 1434. Of this sample, cognitive test score-specific samples ranged from 1122 for CRT to a maximum of 1431 for the BVRT.

### Statistical methods

STATA 18 was used for all analyses (StataCorp, College Station, TX). The first-visit study sample characteristics were reported in terms of PTSD load across lifestyle, health-related, and sociodemographic factors. Differences in continuous, binary, and categorical multi-level covariates were examined using bivariate linear, logistic, and multinomial logit models, in addition to means and proportions. Second, a number of bivariate linear regression models were run in order to evaluate our major hypothesis. The primary exposure in these models was PTSD, and the outcome was cognitive function as evaluated by 13 distinct test scores, with varying degrees of adjustment made for potentially confounding factors.

It was anticipated that the confounders would bias our findings with respect to the associations of PTSD with depressive symptoms and cognitive performance. PTSD score tertiles were compared for a range of categorical variables using bivariate linear and multinomial logistic regression models, with T1 serving as the common referent for the primary tertile predictor variable. In order to develop the linear regression models, possible confounders such as lifestyle, health, and sociodemographic characteristics were sequentially examined. We used multiple imputations for variables to guarantee uniformity in sample size across models. This was achieved using chained Eqs. (5 imputations, 10 iterations), where all continuous variables were centered on their averages and all covariates were employed simultaneously in the estimate procedure, as was the case in earlier research [38, 39].

Firstly, sociodemographic, lifestyle, and health factors, PTSD, depressive symptoms, and cognitive test scores were detailed before and after stratification according to PTSD tertiles, after eliminating HANDLS patients with missing MMSE data. Then, in order to construct a series of linear regression models for PTSD and depressive symptoms as predictors of cognitive test scores, several sets of variables were taken into account. When making covariate adjustments to model 1, considerations such as age, sex, race, poverty status were made, whereas model 2 further adjusted for education and literacy. All the factors from model 2 were included in model 3, along with drug and smoking usage. Health-related variables including comorbidities, HEI, energy, self-rated health, allostatic load, and IMT were added in model 4, which also corrected for those in model 3.

Structural equation models (SM) were used to examine mediating pathways between PTSD and cognitive test scores through depressive symptoms. Cognitive test scores, the CES-D total score, and PTSD were all converted to standardized *z*-scores within the final selected sample. Exogenous factors in each of these models included the following: age, sex, race, poverty status, education, literacy, mean energy intake (kcal/day), HEI-2010, current smoking, current illicit drug use, self-rated health, BMI, comorbidities, allostatic load, IMT mean. All three endogenous variables in the system were predicted by these exogenous covariates. The endogenous cognitive tests were MMSE (which was normalized), CVLT-List A, CVLT-DFR, BVRT, BTA, AF, DS-F, DS-B, CDT, Ln(TRAILS A), Ln(TRAILS B), Card Rotation, and Identical Picture total score. We subsequently used a similar analytic approach from the prior SM to conduct a second SM across IMT mean tertiles. For each SM, we used STATA command *teffects* to help estimate potential outcome means [40–42]; but to test heterogeneity of various path coefficients across IMT tertiles in SM, we used group invariance test (*estat ginvariant*) to obtain Wald tests reflecting differences across groups for each path coefficient [43].

A two-stage Heckman selection procedure was utilized to address sample selectivity, which may have contributed to missingness on exposure and outcome data in comparison to the originally recruited sample, in all models (linear and SM). Using a probit model, we first predicted a selection indicator based on sociodemographic variables. In this instance, those were poverty status, sex, age, and race. An inverse mills ratio (IMR), a function of the probability of selection contingent on certain sociodemographic parameters, was produced by this model. Using regression and SM models, we estimated the main models evaluating the key hypotheses at the second stage. We also included the IMR as an adjustment covariate in all main models [44].

For the main effects and interactions, we predetermined the type I error rate to be 0.05 and 0.10 respectively [45]. Heatmaps, which are pictorial representations of SM models, were also used to demonstrate the mediating path coefficients and various effects (INDIRECT, DIRECT AND TOTAL EFFECTS of PTSD on cognitive test scores through CES-D total score) and their heterogeneity across IMT tertiles, namely PTSD to CES-D path coefficient across various COGN and CES-D to COGN path coefficient.

## Results

### Study sample characteristics by PTSD tertile groups

Table 1 provides summary statistics for up to 1434 study-eligible HANDLS participants, along with other key variables of interest, on their sociodemographic, lifestyle, and health-related traits, PTSD exposure, depressive symptom scores, and cognitive test scores, both overall and according to tertiles of PTSD. The average CES-D scores for all PTSD tertiles were 14.73 (± 0.30). All depressive symptom scores showed differences; however, only CES-D and CES-WB remained statistically significant after adjusting for sociodemographic factors (i.e., age, sex, race, and poverty status). Additionally, participants with less education than high school, those living below poverty, along with current smokers and drug users were more likely to be in the uppermost PTSD tertile even after adjusting for sociodemographic factors compared to their respective counterparts. The uppermost PTSD tertile was also characterized by higher prevalence of cardiovascular disease, a larger number of co-morbid conditions, and poorer dietary quality compared to the lowest PTSD tertile.

**Table 1:** Study sample characteristics: overall and by PTSD tertile groups, HANDLS 2004–2009^a,b^

	Overall	T1	T2	T3	Tertile differences *P*-value	Adjusted tertile differences *P*-value^c^
	Mean ± SE or %	Mean ± SE or %	Mean ± SE or %	Mean ± SE or %		
	*N* = 1434	*N* = 480	*N* = 509	*N* = 445		
**Sociodemographic**						
***Sex***						
Male	43.4	44.6	43.0	42.5	0.018	0.314
Female	56.6	55.4	57.0	57.5	–	–
***Age (years)***						
Mean ± SE	47.38 ± 24.04	47.85 ± 40.99	47.99 ± 42.02	46.17 ± 41.21	< 0.001	< 0.001
***Race/ethnicity***						
White	47.5	43.5	51.5	47.2	0.005	0.002
Black	52.5	56.5	48.5	52.8	–	–
***Poverty status***						
Above	58.4	64.8	62.9	46.3	–	–
Below	41.6	35.2	37.1	53.7	< 0.001	0.001
***Education***						
< High school graduate	5.9	7.3	7.4	7.7	< 0.001	< 0.001
High school graduate	58.0	51.3	54.4	64.2		
≥ High school graduate	36.1	41.4	38.2	28.1	< 0.001	< 0.001
**Lifestyle**						
***Smoking status***						
Never/past smoker	50.6	55.2	53.4	42.4	–	–
Current smoker	49.4	44.8	46.6	57.6	0.027	< 0.001
***Current drug use***						
Never/former	80.0	85.8	80.7	72.9	–	–
Current	20.0	14.3	19.3	27.2	< 0.001	< 0.001
**Health related**						
***Self-rated health***						
Excellent/very good/good	24.5	12.9	23.0	38.7	< 0.001	< 0.001
Fair/poor	37.1	47.5	35.4	27.9	0.274	< 0.001
***Body mass index (kg/m***^***2***^**)**						
Mean ± SE	29.35 ± 19.95	29.02 ± 31.75	30.32 ± 35.19	28.60 ± 36.17	< 0.001	< 0.001
***Healthy Eating Index total score***						
Mean ± SE	42.52 ± 37.12	43.78 ± 66.53	42.72 ± 56.51	40.93 ± 52.06	< 0.001	< 0.001
***Comorbid conditions***^d^						
Mean ± SE	4.24 ± 3.58	4.14 ± 5.96	4.34 ± 6.19	4.23 ± 6.41	< 0.001	< 0.001
***Hypertension***						
No	59.6	60.5	58.2	60.0	–	–
Yes	40.5	39.5	41.8	40.0	< 0.001	< 0.001
***Diabetes status***						
None	70.0	72.1	65.4	72.8	_	_
Prediabetes	16.4	15.3	19.1	14.7	< 0.001	< 0.001
Diabetes	13.6	12.6	15.5	12.5	< 0.001	< 0.001
***Dyslipidemia***						
No	76.3	78.4	74.7	75.8	–	–
Yes	23.7	21.6	25.3	24.2	< 0.001	< 0.001
***Cardiovascular diseases***^e^						
No	84.0	87.8	83.2	80.6	–	–
Yes	16.0	12.2	16.8	19.4	< 0.001	< 0.001
***Lipid-lowering drugs***						
No	88.3	89.5	86.8	88.7	–	–
Yes	11.7	10.5	13.2	11.3	< 0.001	< 0.001
***Allostatic load***						
Mean ± SE	1.81 ± 3.67	1.68 ± 0.5.71	1.89 ± 5.82	1.84 ± 6.85	< 0.001	0.172
***Energy mean***						
Mean ± SE	2043.65 ± 28.46	2086.67 ± 48.12	1988.66 ± 47.79	2060.15 ± 49.15	< 0.001	< 0.001
***WRAT total score***						
Mean ± SE	42.81 ± 20.95	43.87 ± 34.65	43.46 ± 35.13	40.92 ± 37.91	< 0.001	< 0.001
***Depressive symptom scores (mean* ± *SE)***						
CES	14.73 ± 0.30	7.23 ± 0.30	13.10 ± 0.37	24.69 ± 0.53	< 0.001	0.011
CES_DA	4.55 ± 0.13	1.61 ± 0.12	3.77 ± 0.17	8.62 ± 0.25	< 0.001	0.065
CES_IP	1.01 ± 0.04	0.45 ± 0.04	0.88 ± 0.05	1.76 ± 0.07	< 0.001	0.818
CES_SC	6.70 ± 0.12	3.82 ± 0.15	6.46 ± 0.16	10.09 ± 0.19	< 0.001	0.094
CES_WB	9.53 ± 0.07	10.66 ± 0.09	10.01 ± 0.10	7.78 ± 0.14	< 0.001	< 0.001
***Cognitive test scores (mean* ± *SE)***						
Normalized MMSE	76.81 ± 0.42	78.60 ± 0.70	77.46 ± 0.71	74.15 ± 0.76	< 0.001	< 0.001
CVLT-List A	24.60 ± 0.20	25.50 ± 0.32	25.08 ± 0.32	23.15 ± 0.37	< 0.001	< 0.001
CVLT-DFR	7.33 ± 0.09	7.63 ± 0.15	7.58 ± 0.15	6.74 ± 0.17	< 0.001	< 0.001
BVRrot	0.80 ± 0.03	0.79 ± 0.04	0.78 ± 0.04	0.85 ± 0.05	< 0.001	0.008
BTA	6.72 ± 0.06	6.91 ± 0.10	6.72 ± 0.11	6.52 ± 0.11	< 0.001	< 0.001
AF	19.12 ± 0.15	19.32 ± 0.25	19.68 ± 0.25	18.27 ± 0.25	< 0.001	< 0.001
DS-F	7.42 ± 0.06	7.71 ± 0.10	7.58 ± 0.10	6.93 ± 0.11	< 0.001	< 0.001
DS-B	5.70 ± 0.06	5.88 ± 0.10	5.88 ± 0.10	5.29 ± 0.10	< 0.001	< 0.001
CDT	8.81 ± 0.03	8.89 ± 0.05	8.89 ± 0.05	8.65 ± 0.06	< 0.001	< 0.001
Ln(TRAILS A)	3.47 ± 0.01	3.44 ± 0.02	3.46 ± 0.02	3.52 ± 0.02	< 0.001	< 0.001
Ln(TRAILS B)	4.63 ± 0.02	4.54 ± 0.03	4.59 ± 0.03	4.77 ± 0.04	< 0.001	< 0.001
CRT	35.86 ± 0.55	36.75 ± 0.96	36.62 ± 0.90	33.95 ± 1.02	< 0.001	< 0.001
IDP	23.80 ± 0.19	24.63 ± 0.33	23.99 ± 0.33	22.71 ± 0.33	< 0.001	< 0.001
***Intima-media thickness, cm***						
Mean ± SE	0.06904+/-0.00034	0.0693+/- 0.0006	0.06880 +/- 0.0006	0.06906 +/- 0.0006	< 0.001	< 0.001
***PTSD score***						
Mean ± SE	12.46 ± 0.33	1.70 ± 0.07	8.90 ± 0.13	28.13 ± 0.52	< 0.001	< 0.001

*Abbreviations:* AF, Animal Fluency; BTA,Brief Test of Attention; *BVRrot*, Benton Visual Retention Test; *CDT*, Clock Drawing Test; *CRT*, Card Rotation Test; *CES-D*, Center for Epidemiologic Studies-Depression; *CES_DA*, depressive affect; *CES_IP*, interpersonal problems; *CES_SC*, somatic complaints; *CES_WB*, positive affect; *CVLT-List A*, California Verbal Learning Test-List A; *CVLT-DFR*, California Verbal Learning Test-Delay Free Recall; *DS-F*, Digit Span Forward; *DS-B*, Digit Span Backward; *HANDLS*, Healthy Aging in Neighborhoods of Diversity Across the Life Span; *IDP*, Identical Pictures; *MMSE*, mini-mental total score; T1, First Tertile; T2, Second Tertile; T3, Third Tertile; *TRIALS A*, Trail Making Test Part A; *TRIALS B*, Trail Making Test Part B; *SE*, standard error; *WRAT*, Wide Range Achievement Test

^a^Values are means ± SE or column percentages for sample characteristics, overall and across PTSD tertiles

^b^Based on linear or multinomial logit models using PTSD tertile as the main predictor for both continuous and categorical variables, respectively

^c^Adjusted tertile differences included age, sex, race, and poverty status in the model

^d^Comorbid conditions include hypertension, diabetes, dyslipidemia, and cardiovascular diseases

^e^Cardiovascular disease includes atrial fibrillation, angina, coronary artery disease, congestive heart failure, and myocardial infarction

### Linear regression model findings for PTSD and depressive symptom associations in overall sample

Results from linear regression models examining the relationships between 13 cognitive test scores and PTSD and depressive symptoms are shown in Table 2. In model 1, there were significant associations between PTSD and all cognitive test scores, PTSD and all depressive symptom scores including depression sub-scores, and all depressive symptoms sub-scores across each cognitive test score. For the majority of the cognitive tests in models 2, 3, and 4, associations were markedly attenuated. However, the relationship between PTSD and depressive symptoms consistently remained significant across all models, including fully adjusted model 4, which included additional health factors, such as comorbid conditions, HEI-2010, mean energy intake, self-rated health, allostatic load, and IMT mean, in addition to sociodemographic factors. The findings including these covariates confirm the hypothesized relationship between depressive symptoms and PTSD.

**Table 2:** PTSD score, depressive symptoms and 13 cognitive test scores associations based on multiple linear regression models: HANDLS 2004–2009^a,b,c^

	**Model 1**		**Model 2**		**Model 3**		**Model 4**	
	***β* ± SE**	***P***	***β* ± SE**	***P***	***β* ± SE**	***P***	***β* ± SE**	***P***
**X = PTSD vs. Y = Cognitive test scores**								
Normalized MMSE	− 0.15032 ± 0.03139	< 0.001	− 0.04675 ± 0.02716	0.085	− 0.04632 ± 0.02731	0.090	− 0.04534 ± 0.02799	0.105
CVLT-List A	− 0.08997 ± 0.01425	< 0.001	− 0.06283 ± 0.01353	< 0.001	− 0.06175 ± 0.01361	< 0.001	− 0.05826 ± 0.01391	< 0.001
CVLT-DFR	− 0.03387 ± 0.00666	< 0.001	− 0.02267 ± 0.00645	< 0.001	− 0.02194 ± 0.00649	0.001	− 0.02145 ± 0.00663	0.001
BVRT	+ 0.06494 ± 0.01039	< 0.001	+ 0.04540 ± 0.01009	< 0.001	+ 0.04413 ± 0.01014	< 0.001	+ 0.03916 ± 0.01035	< 0.001
BTA	− 0.01064 ± 0.00475	0.025	− 0.00308 ± 0.00462	0.505	− 0.00383 ± 0.00465	0.411	− 0.00185 ± 0.00472	0.695
AF	− 0.04240 ± 0.01123	< 0.001	− 0.02157 ± 0.01090	0.048	− 0.02295 ± 0.01096	0.036	− 0.02145 ± 0.01122	0.056
DS-F	− 0.02755 ± 0.00461	< 0.001	− 0.01542 ± 0.00425	< 0.001	− 0.01689 ± 0.00426	< 0.001	− 0.01741 ± 0.00436	< 0.001
DS-B	− 0.01797 ± 0.00449	< 0.001	− 0.00547 ± 0.00407	0.180	− 0.00590 ± 0.00410	0.150	− 0.00490 ± 0.00420	0.243
CDT	− 0.01068 ± 0.00255	< 0.001	− 0.00741 ± 0.00254	0.004	− 0.00753 ± 0.00256	0.003	− 0.00792 ± 0.00262	0.003
Ln(TRAILS A)	+ 0.00274 ± 0.00184	0.001	+ 0.00162 ± < 0.00183	0.053	+ 0.00164 ± < 0.00184	0.051	+ 0.00128 ± < 0.00185	0.135
Ln(TRAILS B)	+ 0.00817 ± 0.00145	< 0.001	+ 0.00452 ± 0.00135	0.001	+ 0.00446 ± 0.00136	0.001	+ 0.00418 ± 0.00139	0.003
IDP	− 0.06625 ± 0.01255	< 0.001	− 0.04336 ± 0.01212	< 0.001	− 0.04227 ± 0.01217	0.001	− 0.03599 ± 0.01242	0.004
CRT	− 0.13293 ± 0.04021	0.001	− 0.08728 ± 0.03944	0.027	− 0.08513 ± 0.03967	0.032	− 0.08772 ± 0.04065	0.031
**X = PTSD vs. Y = Depressive symptoms**								
CES	+ 0.59068 ± 0.01754	< 0.001	+ 0.57749 ± 0.01768	< 0.001	+ 0.57249 ± 0.01773	< 0.001	+ 0.54190 ± 0.01772	< 0.001
CES_DA	+ 0.24121 ± 0.00786	< 0.001	+ 0.23810 ± 0.00797	< 0.001	+ 0.23593 ± 0.00799	< 0.001	+ 0.22421 ± 0.00805	< 0.001
CES_IP	+ 0.04259 ± 0.00262	< 0.001	+ 0.04067 ± 0.00264	< 0.001	+ 0.04012 ± 0.00265	< 0.001	+ 0.03928 ± 0.00271	< 0.001
CES_SC	+ 0.20623 ± 0.00736	< 0.001	+ 0.20151 ± 0.00743	< 0.001	+ 0.20007 ± 0.00746	< 0.001	+ 0.18788 ± 0.00746	< 0.001
CES_WB	− 0.10064 ± 0.00485	< 0.001	− 0.09722 ± 0.00489	< 0.001	− 0.09637 ± 0.00491	< 0.001	− 0.09053 ± 0.00497	< 0.001
**X = CES vs. Y = Cognitive test scores**								
Normalized MMSE	− 0.18892 ± 0.03535	< 0.001	− 0.05054 ± 0.03080	0.101	− 0.04991 ± 0.03108	0.109	− 0.04896 ± 0.03276	0.135
CVLT-List A	− 0.12528 ± 0.01600	< 0.001	− 0.09076 ± 0.01528	< 0.001	− 0.08965 ± 0.01539	< 0.001	− 0.08849 ± 0.01620	< 0.001
CVLT-DFR	− 0.04917 ± 0.00749	< 0.001	− 0.03449 ± 0.00731	< 0.001	− 0.03366 ± 0.00738	< 0.001	− 0.03484 ± 0.00776	< 0.001
BVRT	+ 0.07922 ± 0.01171	< 0.001	+ 0.05303 ± 0.01146	< 0.001	+ 0.05138 ± 0.01155	< 0.001	+ 0.04291 ± 0.01209	< 0.001
BTA	− 0.01822 ± 0.00541	0.001	− 0.00906 ± 0.00529	0.087	− 0.01008 ± 0.00533	0.058	− 0.00705 ± 0.00556	0.205
AF	− 0.06486 ± 0.01259	< 0.001	− 0.03720 ± 0.01233	0.003	− 0.03939 ± 0.01242	0.002	− 0.03738 ± 0.01304	0.004
DS-F	− 0.02770 ± 0.00518	< 0.001	− 0.01180 ± 0.00481	0.014	− 0.01382 ± 0.00484	0.004	− 0.01430 ± 0.00508	0.005
DS-B	− 0.02417 ± 0.00504	< 0.001	− 0.00722 ± 0.00461	0.117	− 0.00789 ± 0.00465	0.090	− 0.00632 ± 0.00489	0.196
CDT	− 0.01296 ± 0.00288	< 0.001	− 0.00865 ± 0.00288	0.003	− 0.00883 ± 0.00291	0.002	− 0.00956 ± 0.00306	0.002
Ln(TRAILS A)	+ 0.00345 ± < 0.00195	< 0.001	+ 0.00200 ± < 0.00195	0.035	+ 0.00204 ± < 0.00195	0.032	+ 0.00131 ± 0.00100	0.189
Ln(TRAILS B)	+ 0.01043 ± 0.00163	< 0.001	+ 0.00557 ± 0.00153	< 0.001	+ 0.00551 ± 0.00154	< 0.001	+ 0.00510 ± 0.00161	0.002
IDP	− 0.07816 ± 0.01431	< 0.001	− 0.04917 ± 0.01387	< 0.001	− 0.04765 ± 0.01399	0.001	− 0.03559 ± 0.01474	0.016
CRT	− 0.14588 ± 0.04570	0.001	− 0.08082 ± 0.04512	0.074	− 0.07800 ± 0.04554	0.087	− 0.07808 ± 0.04796	0.104
**X = CES_DA vs. Y = Cognitive test scores**								
Normalized MMSE	− 0.35993 ± 0.08243	< 0.001	− 0.10767 ± 0.07102	0.130	− 0.10836 ± 0.07167	0.131	− 0.10601 ± 0.07471	0.156
CVLT-List A	− 0.26834 ± 0.03719	< 0.001	− 0.20514 ± 0.03516	< 0.001	− 0.20284 ± 0.03540	< 0.001	− 0.19844 ± 0.03691	< 0.001
CVLT-DFR	− 0.10721 ± 0.01732	< 0.001	− 0.08033 ± 0.01673	< 0.001	− 0.07857 ± 0.01687	< 0.001	− 0.08012 ± 0.01757	< 0.001
BVRT	+ 0.15577 ± 0.02727	< 0.001	+ 0.10774 ± 0.02639	< 0.001	+ 0.10390 ± 0.02658	< 0.001	+ 0.08593 ± 0.02755	0.002
BTA	− 0.03795 ± 0.01249	0.002	− 0.02252 ± 0.01210	0.063	− 0.02479 ± 0.01219	0.042	− 0.01798 ± 0.01262	0.154
AF	− 0.10975 ± 0.02932	< 0.001	− 0.05903 ± 0.02840	0.038	− 0.06397 ± 0.02860	0.025	− 0.05833 ± 0.02974	0.050
DS-F	− 0.04891 ± 0.01206	< 0.001	− 0.01938 ± 0.01106	0.080	− 0.02385 ± 0.01112	0.032	− 0.02478 ± 0.01157	0.032
DS-B	− 0.04546 ± 0.01169	< 0.001	− 0.01433 ± 0.01057	0.175	− 0.01584 ± 0.01066	0.138	− 0.01220 ± 0.01111	0.273
CDT	− 0.02539 ± 0.00668	< 0.001	− 0.01738 ± 0.00663	0.009	− 0.01790 ± 0.00669	0.007	− 0.01898 ± 0.00696	0.007
Ln(TRAILS A)	0.00696 ± 0.00220	0.002	+ 0.00430 ± 0.00217	0.048	+ 0.00424 ± 0.00219	0.053	+ 0.00277 ± 0.00227	0.223
Ln(TRAILS B)	+ 0.02302 ± 0.00378	< 0.001	+ 0.01428 ± 0.00351	< 0.001	+ 0.01421 ± 0.00354	< 0.001	+ 0.01335 0.00367	< 0.001
IDP	− 0.16582 ± 0.03304	< 0.001	− 0.11643 ± 0.03170	< 0.001	− 0.11323 ± 0.03198	< 0.001	− 0.09154 ± 0.03331	0.006
CRT	− 0.24162 ± 0.10572	0.022	− 0.13662 ± 0.10335	0.186	− 0.13217 ± 0.10432	0.205	− 0.12969 ± 0.10878	0.233
**X = CES_IP vs. Y = Cognitive test scores**								
Normalized MMSE	− 1.80342 ± 0.28919	< 0.001	− 0.80718 ± 0.25123	0.001	− 0.82085 ± 0.25252	0.001	− 0.82589 ± 0.25489	0.001
CVLT-List A	− 0.87279 ± 0.13380	< 0.001	− 0.63810 ± 0.12676	< 0.001	− 0.63101 ± 0.12737	< 0.001	− 0.60715 ± 0.12829	< 0.001
CVLT-DFR	− 0.42668 ± 0.06144	< 0.001	− 0.32827 ± 0.05957	< 0.001	− 0.32370 ± 0.05996	< 0.001	− 0.31929 ± 0.06038	< 0.001
BVRT	+ 0.55673 ± 0.09681	< 0.001	+ 0.37241 ± 0.09402	< 0.001	+ 0.36173 ± 0.09446	< 0.001	+ 0.33338 ± 0.09490	< 0.001
BTA	− 0.17330 ± 0.04502	< 0.001	− 0.10304 ± 0.04391	0.019	− 0.10982 ± 0.04413	0.013	− 0.09915 ± 0.04434	0.026
AF	− 0.50463 ± 0.10400	< 0.001	− 0.31377 ± 0.10120	0.002	− 0.32920 ± 0.10173	0.001	− 0.32295 ± 0.10254	0.002
DS-F	− 0.21349 ± 0.04297	< 0.001	− 0.09654 ± 0.03969	0.015	− 0.10992 ± 0.03982	0.006	− 0.11051 ± 0.04007	0.006
DS-B	− 0.21745 ± 0.04166	< 0.001	− 0.09601 ± 0.03799	0.012	− 0.10082 ± 0.03810	0.008	− 0.09621 ± 0.03854	0.013
CDT	− 0.11461 ± 0.02369	< 0.001	− 0.08376 ± 0.02362	< 0.001	− 0.08562 ± 0.02374	< 0.001	− 0.08742 ± 0.02391	< 0.001
Ln(TRAILS A)	+ 0.02519 ± 0.00781	0.001	+ 0.01428 ± 0.00778	0.067	+ 0.01374 ± 0.00780	0.078	+ 0.01218 ± 0.00782	0.119
Ln(TRAILS B)	+ 0.08127 ± 0.01350	< 0.001	+ 0.04704 ± 0.01258	< 0.001	+ 0.04679 ± 0.01266	< 0.001	+ 0.04556 ± 0.01275	< 0.001
IDP	− 0.55474 ± 0.11902	< 0.001	− 0.35818 ± 0.11455	0.002	− 0.34892 ± 0.11521	0.003	− 0.31463 ± 0.11563	0.007
CRT	− 1.49888 ± 0.38316	< 0.001	− 0.99170 ± 0.37736	0.009	− 0.98148 ± 0.37960	0.010	− 0.98067 ± 0.38220	0.010
**X = CES_SC vs. Y = Cognitive test scores**								
Normalized MMSE	− 0.37389 ± 0.09086	< 0.001	− 0.05693 ± 0.07868	0.469	− 0.05169 ± 0.07917	0.514	− 0.04156 ± 0.08330	0.618
CVLT-List A	− 0.25536 ± 0.04134	< 0.001	− 0.16919 ± 0.03934	< 0.001	− 0.16579 ± 0.03955	< 0.001	− 0.15703 ± 0.04159	< 0.001
CVLT-DFR	− 0.09838 ± 0.01931	< 0.001	− 0.06121 ± 0.01880	0.001	− 0.05896 ± 0.01893	0.002	− 0.05970 ± 0.01991	0.003
BVRT	+ 0.17057 ± 0.03017	< 0.001	+ 0.10791 ± 0.02937	< 0.001	+ 0.10384 ± 0.02954	< 0.001	+ 0.07965 ± 0.03085	0.010
BTA	− 0.03596 ± 0.01393	0.010	− 0.01283 ± 0.01360	0.346	− 0.01462 ± 0.01366	0.285	− 0.00685 ± 0.01429	0.632
AF	− 0.14944 ± 0.03237	< 0.001	− 0.08323 ± 0.03158	0.008	− 0.08692 ± 0.03174	0.006	− 0.07982 ± 0.03325	0.017
DS-F	− 0.06175 ± 0.01332	< 0.001	− 0.02656 ± 0.01228	0.031	− 0.03031 ± 0.01233	0.014	− 0.03036 ± 0.01293	0.019
DS-B	− 0.05037 ± 0.01297	< 0.001	− 0.01138 ± 0.01179	0.334	− 0.01254 ± 0.01187	0.291	− 0.00794 ± 0.01247	0.525
CDT	− 0.02271 ± 0.00740	0.002	− 0.01243 ± 0.00738	0.092	− 0.01248 ± 0.00742	0.093	− 0.01320 ± 0.00781	0.091
Ln(TRAILS A)	+ 0.00797 ± 0.00243	0.001	+ 0.00469 ± 0.00242	0.053	+ 0.00494 ± 0.00243	0.042	+ 0.00296 ± 0.00253	0.242
Ln(TRAILS B)	+ 0.02154 ± 0.00419	< 0.001	+ 0.01009 ± 0.00391	0.010	+ 0.00985 ± 0.00394	0.012	+ 0.00851 ± 0.00411	0.039
IDP	− 0.16410 ± 0.03696	< 0.001	− 0.09117 ± 0.03573	0.011	− 0.08711 ± 0.03595	0.016	− 0.05006 ± 0.03791	0.187
CRT	− 0.35439 ± 0.11743	0.003	− 0.019513 ± 0.11587	0.092	− 0.18672 ± 0.11649	0.109	− 0.17973 ± 0.12295	0.144
**X = CES_WB vs. Cognitive test scores**								
Normalized MMSE	+ 0.69316 ± 0.15038	< 0.001	− 0.16851 ± 0.13019	0.196	+ 0.16249 ± 0.13093	0.215	+ 0.14801 ± 0.13521	0.274
CVLT-List A	+ 0.42334 ± 0.06835	< 0.001	+ 0.29711 ± 0.06474	< 0.001	+ 0.29237 ± 0.06499	< 0.001	+ 0.27891 ± 0.06696	< 0.001
CVLT-DFR	+ 0.13928 ± 0.03213	< 0.001	+ 0.08698 ± 0.03104	0.005	+ 0.08385 ± 0.03117	0.007	+ 0.08311 ± 0.03209	0.010
BVRT	− 0.28969 ± 0.04995	< 0.001	− 0.18893 ± 0.04856	< 0.001	− 0.18296 0.04875	< 0.001	− 0.14757 ± 0.05008	0.003
BTA	+ 0.05692 ± 0.02297	0.013	+ 0.02377 ± 0.02232	0.287	0.02727 ± 0.02245	0.225	+ 0.01644 ± 0.02302	0.475
AF	+ 0.25989 ± 0.05362	< 0.001	+ 0.15555 ± 0.05222	0.003	+ 0.16110 ± 0.05241	0.002	+ 0.14808 ± 0.05405	0.006
DS-F	+ 0.11009 ± 0.02213	< 0.001	+ 0.04737 0.02042	0.021	+ 0.05347 ± 0.02048	0.009	+ 0.05229 ± 0.02111	0.013
DS-B	+ 0.08878 ± 0.02157	< 0.001	+ 0.02428 0.01959	0.215	+ 0.02604 ± 0.01969	0.186	+ 0.02043 ± 0.02029	0.314
CDT	+ 0.05601 ± 0.01221	< 0.001	+ 0.03964 ± 0.01218	0.001	+ 0.03993 ± 0.01223	0.001	+ 0.04174 ± 0.01262	0.001
Ln(TRAILS A)	− 0.01035 ± 0.00404	0.010	− 0.00458 ± 0.00401	0.254	− 0.00484 ± 0.00402	0.229	− 0.00207 ± 0.00412	0.616
Ln(TRAILS B)	− 0.03017 ± 0.00699	< 0.001	− 0.01115 ± 0.00650	0.086	− 0.01072 ± 0.00653	0.101	− 0.00804 ± 0.00672	0.231
IDP	+ 0.25585 ± 0.06132	< 0.001	+ 0.14141 ± 0.05910	0.017	+ 0.13553 ± 0.05937	0.023	+ 0.08615 ± 0.06108	0.159
CRT	+ 0.46003 ± 0.19411	0.018	+ 0.19697 ± 0.19101	0.303	+ 0.18182 ± 0.19204	0.344	+ 0.17225 ± 0.19815	0.385

*Abbreviations:* AF, Animal Fluency; *BTA, Brief Test of Attention*; *BVRrot*, Benton Visual Retention Test; *CDT*, Clock Drawing Test; *CES-D*, Center for Epidemiologic Studies-Depression; *CES_DA*, depressive affect; *CES_IP*, interpersonal problems; *CES_SC*, Somatic complaints; *CES_WB*, positive affect; *CRT*, Card Rotation Test; *CVLT-List A*, California Verbal Learning Test-List A; *CVLT-DFR*, California Verbal Learning Test-Delay Free Recall; *DS-F*, Digit Span Forward; *DS-B*, Digit Span Backward; *HANDLS*, Healthy Aging in Neighborhoods of Diversity Across the Life Span; *IDP*, Identical Picture; *IMR*, inverse mills ratio; *IMT*, intima-media thickness; *MMSE*, mini-mental total score; *SE*, standard error; *TRIALS A*, Trail Making Test Part A; *TRIALS B*, Trail Making Test Part B

^a^Values are adjusted regression coefficients for PTSD score (raw unstandardized score) or CES-D scores (raw unstandardized scores), with the final outcome being each of 13 cognitive test scores (raw, unstandardized scores)

^b^Model 1 includes age, race, sex, and poverty status and IMR. Model 2 includes covariates in model 1 plus education and WRATtotal. Model 3 includes all covariates of models 1 and 2 plus smoking and drug use. Model 4 includes all covariates of previous models plus comorbidity, HEI-2010, mean energy intake, self-rated health, allostatic load, and IMT

^c^Based on linear models with outcomes being either CES-D total score or each of the 13 cognitive test scores. Values are regression coefficient estimates with standard errors and *P*-values (*β* ± SE, *P*) for PTSD as the main predictor for each of the 13 cognitive test scores or CES-D; or regression coefficient estimates with SE for CES-D (and sub-score) as predicting each of the 13 cognitive test scores. Note that total and sub-scores of CES-D were entered separately in each model with each of the 13 cognitive test scores as alternative outcomes

### Structural equation model findings: PTSD vs. cognitive test scores through CES-D total score 

The findings of the SM, which tested the direct effect (DE) and indirect effect (IE) of PTSD on cognitive function using CES-D as a potential mediator, are shown in Table 3. Upon concentrating on models possessing significant total effects (TE), it was shown that the IE was notably significant just for the key mediating variables in the whole sample such as CVLT-DFR (IE = − 0.06786 ± 0.02202, *P* < 0.05; TE = − 0.08398 ± 0.02654, *P* < 0.05) and CVLT-List A (IE = − 0.07625 ± 0.02140, *P* < 0.05; TE = − 0.10734 ± 0.02571). The majority of other models (e.g., BVRT, DS-F, CDT, Ln(TRAILS B), CRT, and IDP) that showed a substantial TE or DE of PTSD on cognitive function suggested the involvement of a different mechanism that did not include CES-D.

**Table 3:** Association of PTSD with each of 13 cognitive test scores, through CES-D total score, across 5 imputations, based on structural equations models (SM): HANDLS 2004–2009^a,b^

Cognitive tests	TE ± SE	*P*_TE_	DE ± SE	*P*_DE_	IE ± SE	*P*_IE_
Normalized MMSE	− 0.03726 ± 0.02220	0.093	− 0.02544 ± 0.02861	0.374	− 0.01182 ± 0.01797	0.511
CVLT-List A	− 0.10734 ± 0.02571	< 0.001	− 0.03109 ± 0.03324	0.350	− 0.07625 ± 0.02140	< 0.001
CVLT-DFR	− 0.08398 ± 0.02654	0.002	− 0.01612 ± 0.03432	0.639	− 0.06786 ± 0.02202	0.002
BVRT	0.09707 ± 0.02540	< 0.001	0.06567 ± 0.03274	0.045	0.03141 ± 0.02071	0.129
BTA	− 0.01167 ± 0.02674	0.663	0.01861 ± 0.03463	0.591	− 0.03028 ± 0.02071	0.170
AF	− 0.04769 ± 0.02674	0.062	− 0.00436 ± 0.03287	0.894	− 0.04333 ± 0.02071	0.037
DS-F	− 0.10055 ± 0.02471	< 0.001	− 0.09200 ± 0.03186	0.004	− 0.00855 ± 0.02011	0.671
DS-B	− 0.02797 ± 0.02384	0.241	− 0.01395 ± 0.03076	0.650	− 0.01402 ± 0.01946	0.471
CDT	− 0.08284 ± 0.02705	0.002	− 0.04770 ± 0.03481	0.171	− 0.03515 ± 0.02197	0.110
Ln(TRAILS A)	0.03783 ± 0.02504	0.131	0.02792 ± 0.03237	0.388	0.00991 ± 0.02052	0.629
Ln(TRAILS B)	0.06983 ± 0.02296	0.002	0.03940 ± 0.02966	0.184	0.03043 ± 0.01882	0.106
CRT	− 0.05995 ± 0.02750	0.029	− 0.05256 ± 0.03602	0.144	− 0.00739 ± 0.02326	0.751
IDP	− 0.06772 ± 0.02331	0.004	− 0.05374 ± 0.03036	0.077	− 0.01398 ± 0.01946	0.473

^a^Values are TE, DE, and IE from SM, with the final outcome being each of the 13 cognitive test scores, the main exposure being PTSD, and the potential mediator being CES-D total score. All are standardized *z*-scores

^b^SM models adjusted for all exogenous variables listed in model 3 of Table 2, namely age, race, sex, poverty status, education, WRAT total, smoking and drug use, comorbid, HEI-2010, mean energy intake, self-rated health, allostatic load, IMT, and the IMR

*AF*, Animal Fluency; *BTA, Brief Test of Attention*; *BVRrot*, Benton Visual Retention Test; *CDT*, Clock Drawing Test; *CES-D*, Center for Epidemiologic Studies-Depression; *CRT*, Card Rotation Test; *CVLT-List A*, California Verbal Learning Test-List A; *CVLT-DFR*, California Verbal Learning Test-Delay Free Recall; *DE*, direct effect; *DS-F*, Digit Span Forward; *DS-B*, Digit Span Backward; *HANDLS*, Healthy Aging in Neighborhoods of Diversity Across the Life Span; *IDP*, Identical Picture; *IE*, indirect effect; *IMR*, inverse mills ratio; *IMT*, intima-media thickness; *MMSE*, mini-mental total score; *SE*, standard error; *TE*, total effect; *TRIALS A*, Trail Making Test Part A; *TRIALS B*, Trail Making Test Part B

**Fig. 2:**
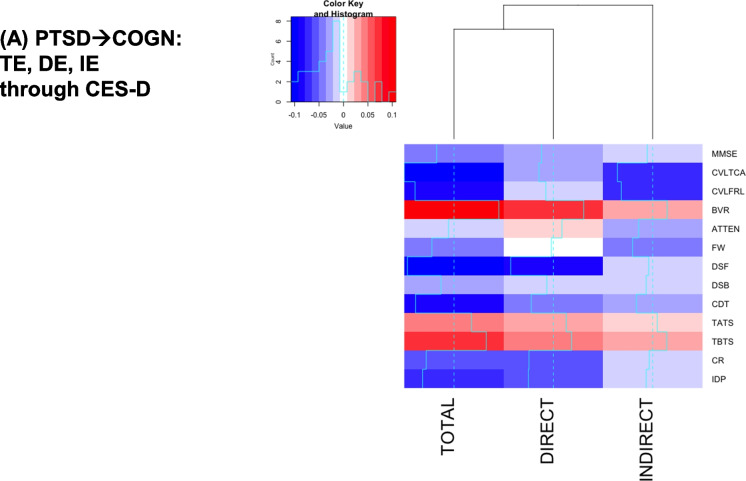

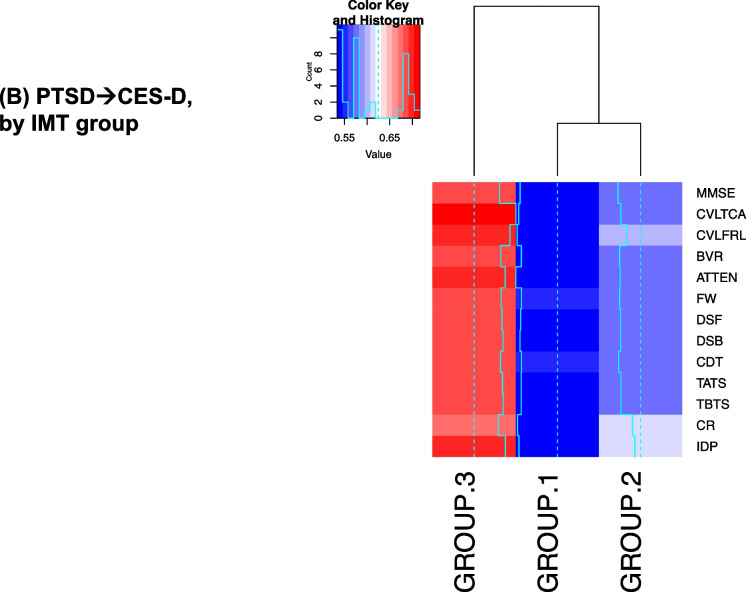

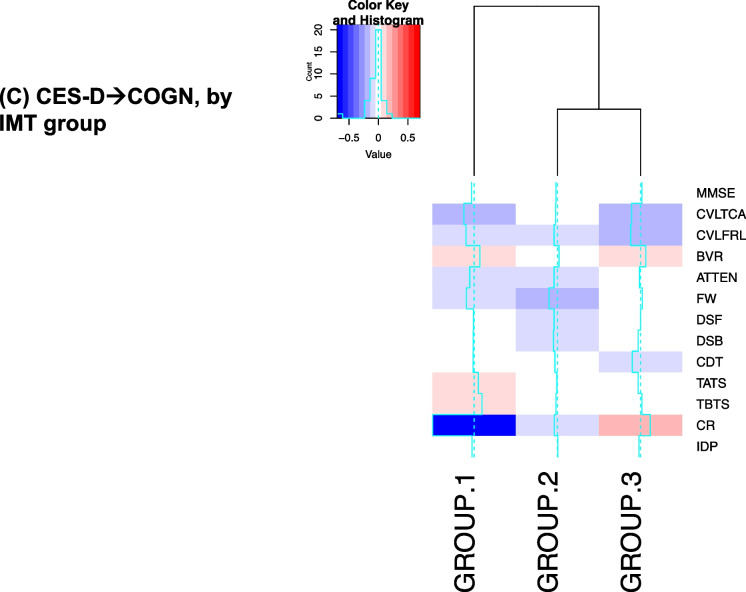
Mediating pathway between PTSD and COGN through CES-D test score and heterogeneity through IMT (Groups 1, 2 and 3, representing each IMT tertile): HANDLS 2004–2009. Abbreviations: ATTEN=Brief Test of Attention; BVR=Benton Visual Retention Test; CDT, Clock Drawing Test; CES-D, Center for Epidemiologic Studies-Depression; DIRECT, Direct Effect, COGN, cognitive performance test score; CR, Card Rotation; CVLTCA, California Verbal Learning Test, Part A; CVLTFRL, California Verbal Learning Test, Free Recall Long; DSB=Digits Span Backward Test; DSF, Digits Span Forward Test; FW, Animal Fluency Test; HANDLS, Healthy Aging in Neighborhoods of Diversity across the Life Span; IDP, Identical Pictures; IMT, intima-media thickness; INDIRECT, Indirect Effect; MMSE, Mini-Mental State Exam; PTSD, post-traumatic stress disorder; TATS, Trails A Test, seconds; TBTS, Trails B Test, seconds; TOTAL, Total Effect; v1, visit 1

Results from a SM testing CES-D as a possible mediator between PTSD and cognitive function are displayed in Fig. 2A. According to our findings, there was an inverse association between the TE of PTSD on cognitive function in the total sample. In the TE, DE, and IE groups, BVRT, Ln(TRAILS A), and Ln(TRAILS B) were all consistently larger than 0.

Figure 2B and C displays the findings from a SM that we used to examine, across IMT mean tertiles, the pathway from PTSD to CES-D and between CES-D and COGN, within the mediating pathway (PTSD to CES-D to COGN). There was significant heterogeneity across IMT tertiles (Groups 1, 2 and 3) in the PTSD to CES-D path coefficients, which was consistent for all cognitive test scores, based on group invariance analysis. More specifically, the association between PTSD and CES-D was stronger at higher IMT tertiles. Detailed numerical findings for Fig. 2(A–C) are presented as a supplementary datasheets 1–7. All outputs, including results datasets used to generate heatmaps, along with Stata and R scripts are stored on GitHub: baydounm/HANDLS_PTSD_CESD_COGN (github.com).

## Discussion

The present study aimed to understand the associations between PTSD, depressive symptoms, and cognitive functioning in a nationally representative sample of urban adults. Linear regression models found that PTSD was strongly related to depressive symptoms, at various levels of adjustment for potential confounders (sociodemographic, lifestyle, and health related factors). Among cognitive test scores in SM, only CVLT-DFR and CVLT-List A were inversely associated with PTSD score, with a TE and IE, indicating that depressive symptoms significantly mediated the potential association of PTSD with poorer verbal memory. Most other cognitive test scores showed a substantial TE or DE of PTSD on cognitive function, suggesting the involvement of a different mechanism that did not include CES-D. At higher IMT tertiles, PTSD score was more strongly associated with the total CES-D score.

### PTSD and cognitive function

PTSD was associated with worse verbal learning and memory, and this association was mediated by greater severity of depressive symptoms. This finding aligns with prior work showing that PTSD is associated with cognitive dysfunction, particularly in verbal learning and memory [6, 8, 46]. It is also consistent with results of neuroimaging studies, which have revealed that memory-relevant brain regions such as the hippocampus are implicated in cognitive impairments in PTSD [9]. Putative mechanisms linking PTSD to decrements in verbal learning and memory include reduced hippocampal volume, hyperarousal symptoms, cognitive interference, and fragmented memory organization [46]. The finding that greater severity of depressive symptoms mediated the association between PTSD symptoms and verbal learning and memory extends prior work demonstrating associations between depression severity and cognitive decline [10, 11]. It is further consistent with results of the Survey of Health, Ageing, and Retirement in Europe, which found that greater severity of PTSD symptoms was associated with greater severity of depressive symptoms, which was in turn linked to cognitive decline [47]. Taken together, these results suggest that PTSD may have a particularly deleterious effect on verbal learning and memory among middle-aged adults, and that greater severity of depressive symptoms may mediate this association.

### PTSD and depressive symptoms

PTSD is a mental health condition that predominantly affects people in all age groups across the lifespan, with onset age being young and middle adulthood. Research also has shown that PTSD has an impact on mood and affect; hence, it is connected to depressive symptoms [16, 17]. In addition, other researchers have found that there is a clear connection between mood disorders, such as depression and PTSD, and cognitive dysfunction. In a systematic review of ten articles, researchers found that those individuals with PTSD and depression are more likely to relate to low motivation, symptoms of major depressive disorder, and poor health behaviors among older adults engaging in exercise programs [17]. Additionally, in a meta-analysis of 61 articles, researchers found that there is a small, albeit significant, association between emotional symptoms and cognitive test performance [48]. Researchers also found that cognitive dysfunction was not only associated with depression but also was associated with PTSD among those individuals who experienced traumatic brain injury.

### Depressive symptoms and cognitive function

A systematic review found that individuals with diabetes and depression experience greater cognitive decline and higher dementia risk, with the potential impact of antidepressant treatment remaining unclear [14]. Another meta-analysis of 16,806 participants found clinical depression and subthreshold depressive symptoms linked to cognitive control deficits, highlighting the need for clinician screening in depression patients [15]. A recent study specifically examined the correlation between social activity, depressive symptoms, and cognitive function among older adults, using data from the Korean Longitudinal Study of Aging. Results show social activity negatively impacts depressive symptoms, while depressive symptoms affect cognitive function [49]. Liang and colleagues detected a bidirectional relationship between post-stroke depressive symptoms and cognitive impairment in 610 ischemic stroke patients, suggesting interventions to improve cognitive function may improve cognition and mood [50]. Another study conducted among older adults in India also concluded that depressed older adults had higher odds of cognitive impairment, particularly in rural areas [13]. The study suggests diagnosing and treating LLD in later life may have significant health implications [13]. Treatment is recommended under a cognitive neurologist or geriatric psychiatrist [13]. These findings were corroborated by another study carried out in Mexico, which found that depression was linked to lower performance in several cognitive domains [51]. It also found that despite improvements in symptoms, formerly depressed individuals still perform worse on cognitive tasks, suggesting researchers should consider depression history [51]. The adverse association between depressive symptoms and cognition was also found in two other large studies in China and Korea [52, 53].

### IMT in relation to PTSD, depressive symptoms, and cognitive function

IMT has previously been associated with PTSD and depressive symptoms. In a recent study of middle-aged women (*n* = 274; mean age = 59.03), higher PTSD scores were associated with larger IMT [18]. Furthermore, in women who were *APO4* carriers, PTSD symptoms were associated with lower cognitive performance specifically for attention and working memory, semantic fluency, perceptual speed, and processing speed [18]. PTSD was associated with IMT in Vietnam War-era veterans [21]. IMT was associated with depressive symptoms in a community cohort of older adults in China [19] and police officers; but this effect was mediated by hypertension [20]. A recent meta-analysis that included 13 different studies of individuals with depression and control individuals (4466 and 21,635, respectively) reported that individuals with depression had significantly larger IMT [54].

For IMT and cognition, a recent meta-analysis of 19 cross-sectional and 15 longitudinal studies with a total of 50,779 individuals reported a small but significant negative association between IMT and cognitive function in cross-sectional studies [55]. This relationship was not observed in longitudinal analyses after adjusting for covariates in cross-sectional studies [56\. In another meta-analysis, larger IMT was associated with risk for mild cognitive impairment, but this analysis did show considerable between study heterogeneity [56]. In a racially diverse cohort from the Bogalusa Heart Study, participants with IMT in the > 50 percentile had lower global cognitive scores, which was independent of cardiovascular risk factors but was buffered through education [57]. In the ELSA-Brazil study, higher IMT was associated with lower cognitive function, and this association was stronger in Whites versus Black participants [58]. Another recent study reported that IMT was associated with depression and cognition in postmenopausal women [59]. Collectively, these studies indicate relationships between IMT and PTSD, depressive symptoms and cognition, but study heterogeneity suggests the importance of examining these relationships in additional studies, especially in diverse populations.

### Strengths and limitations

Our study has several notable strengths. It is one of the largest studies of racially and socio-economically diverse urban adults examining associations between PTSD, depressive symptoms, and cognition in a comprehensive manner, while testing moderation by IMT. The study also included a large battery of cognitive test scores spanning a comprehensive set of domains. A widely recognized measure of depressive symptoms (CES-D) was used, and advanced multivariable techniques were utilized, including SM, which allowed testing mediation by depressive symptoms in the association between PTSD and cognition. Heterogeneity across IMT was tested while adjusting for potential confounders as well as potential sample selectivity. Nevertheless, our study was limited in several ways. First, the cross-sectional design precluded ascertainment of temporality, which highlights that future studies should also include repeat measurement of the cognitive performance tests, among others, to assess the association of PTSD on cognitive decline through depressive symptoms at one point as well as trajectories in depressive symptoms. Second, even though major potentially confounding variables were adjusted for, including socio-demographic, socio-economic, lifestyle, and health-related factors, residual confounding cannot be ruled out.

## Conclusions

In summary, this study provides some evidence that there is an association between PTSD and cognitive functioning among middle-aged urban adults, which was partially mediated through depressive symptoms in the domain of verbal memory, with IMT enhancing the association between PTSD and depressive symptoms. These findings may suggest that, pending interventions including randomized controlled trials, reducing depressive symptoms may alleviate the adverse effect of PTSD on verbal memory and that reducing IMT may attenuate the effect of PTSD on depressive symptoms. Nevertheless, our findings require replication in other cross-sectional and longitudinal studies of comparable populations before interventions are recommended.

## Supplementary Information

Below is the link to the electronic supplementary material.

Supplementary file1 (DOCX 54 KB)

Supplementary file2 (XLSX 55 KB)

## Data Availability

The study protocol (09-AG-N248) received approval from the National Institute on Environmental Health Sciences’ Institutional Review Board (IRB) of the National Institutes of Health (NIH). Upon request, data can be made available to researchers with approved proposals, after they have agreed to confidentiality as required by our IRB. Policies are publicized on https://handls.nih.gov. Data access requests can be sent to principal investigators (PI) or the study manager, Jennifer Norbeck at norbeckje@mail.nih.gov. These data are owned by the National Institute on Aging at the NIH. The PIs have made those data restricted to the public for two main reasons: “(1) The study collects medical, psychological, cognitive, and psychosocial information on racial and poverty differences that could be misconstrued or willfully manipulated to promote racial discrimination; and (2) Although the sample is fairly large, there are sufficient identifiers that the PIs cannot guarantee absolute confidentiality for every participant as we have stated in acquiring our confidentiality certificate.”

## Abbreviations

ACASI: Audio computer-assisted self-interviewing
AF: Animal Fluency test
BVRT: Benton Visual Retention Test
CDT: Clock Drawing Test
CES-D: Center for Epidemiologic Studies-Depression scale
COGN: Cognition
CRT: Card Rotation Test
CVLT: = California Verbal Learning Test
DE: Direct effect
DFR: Delayed Free Recall
DS-F: Digit Span Forward
DS-B: Digit Span Backwards
HANDLS: Healthy Aging in Neighborhoods of Diversity across the Life Span study
HEI-2010: 2010 Healthy Eating Index
IDP: Identical Pictures
IE: Indirect effect
IMR: Inverse mills ratio
IMT: Intima-media thickness
IRP: Intramural Research Program
MMSE: Mini-Mental State Examination
MRV: Mobile research vehicle
NIA: National Institute on Aging
PTSD: Post-traumatic stress disorder
SM: Structural equations models
TE: Total effect
TRAILS A: Trail Making Test Part A
TRAILS B: Trail Making Test Part B
WRAT-3: Wide Range Achievement Test, third edition

## Declarations

### Disclaimer

The work was done outside of the tour of duty at the US Department of Veterans Affairs.
