# Supplementary material for: Post-traumatic stress disorder, depressive symptoms, and cognitive function among middle-aged urban adults: Healthy Aging in Neighborhoods of Diversity across the Life Span study
Source: GeroScience. 2025 Sep 15;48(3):3275–93. doi: 10.1007/s11357-025-01825-0 (PMC13356020; doi:10.1007/s11357-025-01825-0)
Supplement: Supplementary file 1 — Supplementary file1 (DOCX 54 KB) [file 11357_2025_1825_MOESM1_ESM.docx]

**Supplemental Materials**

**Supplemental Material 1 – Cognitive Tests:**

*Mini-Mental State Examination (MMSE)*
The MMSE (Folstein et al., 1975) is a cognitive screening tool utilized to assess global cognitive performance, focusing on orientation, concentration, language, immediate and short-term memory, and constructional praxis. The highest score is 30. Higher numbers indicate improved or superior cognitive performance.
*California Verbal Learning Test (CVLT)*The California Verbal Learning Test (CVLT), developed by Delis et al. in 1988, has a 16-item word list (Delis et al., 1988). A modified version of the CVLT was employed for three learning trials rather than five. Cued recall was not utilized at all. The CVLT outcome variables employed to evaluate verbal learning and memory were the total correct score for List A (learning) and the long-delay free recall score for List A (memory). For learning and memory, there existed two scoring ranges: 0 to 48 and 0 to 16. Elevated scores indicate superior language acquisition and memory retention. A comprehensive description of CVLT is available in other sources (Delis et al., 1988).
*Benton Visual Retention Test (BVRT)*Nonverbal memory and visuoconstructional abilities were assessed using the BVRT (Benton, 1974). Administration A employed form D. According to the BVRT criteria, two experienced examiners reconciled scoring discrepancies; nevertheless, if they could not reach a consensus, a research psychologist assigned the score. The examiners endeavored to resolve any inconsistencies in scoring. The outcome variable was the total number of mistakes; increased values negatively impacted visual memory scores.
*The Digit Span Forward and Backward (DS-F and DS-B)* evaluates working memory and attention, which are the primary components of executive function, as part of the Wechsler Adult Intelligence Scale, Revised (Wechsler, 1981). The tests were administered in accordance with the handbook's guidelines. The dependent variable was the overall score, representing the total number of right responses for each examination.
*Category Fluency*In the fluency test category (Morris et al., 1989; Morris et al., 1988), participants are allotted 60 seconds to generate as many distinct animals as possible, serving as an assessment of semantic verbal fluency. Elevated scores indicate superior category fluency. The outcome variable was the total count of correctly created words, excluding incursions and preservations.
*Brief Test of Attention (BTA)*The examiner can administer a maximum of 10 trials consisting of letters and numerals (4-18 items) in the BTA, a test assessing divided auditory attention, with each trial progressively increasing in duration (Schretlen et al., 1996). Only one examination was administered, consisting of a numerical component. Participants were to record the quantity of repeated values, while the count of letters read during each trial was to be ignored. To prevent counting figures, they were additionally instructed to maintain their hands in clenched fists. The dependent variable was the total count of correct trials.
*Trail Making Tests A and B (TRAILS A and B)*The Trial Making Tests A and B (Reitan, 1992) largely assess attention and executive functioning, respectively. TRIALS B largely encompasses two subcategories of executive function: set-shifting and cognitive control. Both trials encompass assessments of visuomotor scanning and processing velocity. Participants were directed to swiftly draw a line between letters and numbers (TRIALS B) and between consecutive numbers (TRIALS A). They were apprised that time was of the essence. Following the examiner's identification of errors, the participant rectified them. Errors were documented with increased duration. Higher scores indicate lower performance, with completion times reflecting the scores of TRIALS A and B.
*Card Rotation Test*The Card Rotations test (Ekstrom et al., 1976) assesses visuo-spatial abilities by requiring participants to mentally rotate and compare two-dimensional images to ascertain their similarity or dissimilarity. Only part one of this trial was administered, adhering to conventional administration instructions. The total score was calculated by subtracting the number of erroneous questions from the number of correct responses, permitting negative totals.
*Identical Pictures Test*The Identical Pictures test (Ekstrom et al., 1976) assesses psychomotor speed by requiring participants to compare a test object with a set of five comparable objects and select the one that is identical to the test object. Only part one of this trial was conducted, adhering to the conventional administration instructions. The total score was calculated by subtracting the number of erroneous questions from the number of correct responses, which permitted negative totals.
*Clock Drawing Test – Command-Based Clock (CDT)*
The Clock Drawing Test (Rouleau et al., 1992) assessed executive function, memory, and visuospatial skills. Participants are directed to illustrate a clock, include all numerals, and position the hands to eleven minutes past midnight. Performance is contingent upon precise representations of the hands (0-4), numerals (0-4), and clock face (0-2). Superior performance was evidenced by elevated ratings. The potential ratings ranged from 0 to 10. Moreover, individuals who performed poorly on the command component of the examination were required to replicate a clock displaying the time as 10 minutes past eleven.
*Wide Range Achievement Test - Third Edition: Word and Letter Reading Subtest* (WRAT)
The WRAT Word and Letter Reading Subtest (Wilkinson, 1993) is frequently utilized as an indicator of literacy and educational quality. The participants were assigned the job of accurately reading a list of fifty progressively challenging words. Letter reading was employed if the initial five words were spoken inaccurately. The standard protocols were followed for the tan form. The outcome variable utilized was the total count of accurately articulated words.

**Supplemental Material 2 – Depressive Symptoms Questionnaire:**

*Center for Epidemiological Studies Depression Scale (CES-D)*

The 20-item CES-D is an assessment of depressed symptomatology (Nguyen et al., 2004). Participants are requested to document the frequency and severity of their symptoms throughout the past week. The scoring range ranged from 0 to 60. ratings exceeding 16 showed substantial depression symptoms, whereas ratings more than 20 signified clinically significant levels of depressive symptoms.

*Other sources:* (Beydoun et al., 2023a; Beydoun et al., 2024; Beydoun et al., 2016; Beydoun et al., 2020a; Beydoun et al., 2018a; Beydoun et al., 2015; Beydoun et al., 2020b; Beydoun et al., 2018b; Beydoun et al., 2021a; Beydoun et al., 2023b; Beydoun et al., 2020c; Beydoun et al., 2017; Beydoun et al., 2019a; Beydoun et al., 2021b; Beydoun et al., 2019b; Fanelli Kuczmarski et al., 2024; Hossain et al., 2019; Hossain et al., 2020)

**References:**

Benton, A.L., 1974. Revised visual retention test (fifth edition). The Psychological Corportation, New York.

Beydoun, H.A., Beydoun, M.A., Maldonado, A.I., Fanelli-Kuczmarski, M.T., Weiss, J., Evans, M.K., Zonderman, A.B., 2023a. Allostatic Load and Cognitive Function Among Urban Adults in the Healthy Aging in Neighborhoods of Diversity across the Life Span Study. J Alzheimers Dis 92 (2), 425-443.

Beydoun, M.A., Beydoun, H.A., Georgescu, M.F., Maino Vieytes, C.A., Fanelli-Kuczmarski, M.T., Noren Hooten, N., Evans, M.K., Zonderman, A.B., 2024. Plasma homocysteine and longitudinal change in cognitive function among urban adults. J Affect Disord 364, 65-79.

Beydoun, M.A., Canas, J.A., Dore, G.A., Beydoun, H.A., Rostant, O.S., Fanelli-Kuczmarski, M.T., Evans, M.K., Zonderman, A.B., 2016. Serum Uric Acid and Its Association with Longitudinal Cognitive Change Among Urban Adults. J Alzheimers Dis 52 (4), 1415-1430.

Beydoun, M.A., Canas, J.A., Fanelli-Kuczmarski, M.T., Maldonado, A.I., Shaked, D., Kivimaki, M., Evans, M.K., Zonderman, A.B., 2020a. Association of Antioxidant Vitamins A, C, E and Carotenoids with Cognitive Performance over Time: A Cohort Study of Middle-Aged Adults. Nutrients 12 (11).

Beydoun, M.A., Dore, G.A., Canas, J.A., Liang, H., Beydoun, H.A., Evans, M.K., Zonderman, A.B., 2018a. Systemic Inflammation Is Associated With Longitudinal Changes in Cognitive Performance Among Urban Adults. Front Aging Neurosci 10, 313.

Beydoun, M.A., Fanelli-Kuczmarski, M.T., Kitner-Triolo, M.H., Beydoun, H.A., Kaufman, J.S., Mason, M.A., Evans, M.K., Zonderman, A.B., 2015. Dietary antioxidant intake and its association with cognitive function in an ethnically diverse sample of US adults. Psychosom Med 77 (1), 68-82.

Beydoun, M.A., Hossain, S., Beydoun, H.A., Shaked, D., Weiss, J., Evans, M.K., Zonderman, A.B., 2020b. Red Cell Distribution Width Is Directly Associated with Poor Cognitive Performance among Nonanemic, Middle-Aged, Urban Adults. J Nutr 150 (1), 128-139.

Beydoun, M.A., Hossain, S., Fanelli-Kuczmarski, M.T., Beydoun, H.A., Canas, J.A., Evans, M.K., Zonderman, A.B., 2018b. Vitamin D Status and Intakes and Their Association With Cognitive Trajectory in a Longitudinal Study of Urban Adults. J Clin Endocrinol Metab 103 (4), 1654-1668.

Beydoun, M.A., Noren Hooten, N., Beydoun, H.A., Maldonado, A.I., Weiss, J., Evans, M.K., Zonderman, A.B., 2021a. Plasma neurofilament light as a potential biomarker for cognitive decline in a longitudinal study of middle-aged urban adults. Transl Psychiatry 11 (1), 436.

Beydoun, M.A., Noren Hooten, N., Weiss, J., Beydoun, H.A., Georgescu, M., Freeman, D.W., Evans, M.K., Zonderman, A.B., 2023b. GDF15 and its association with cognitive performance over time in a longitudinal study of middle-aged urban adults. Brain Behav Immun 108, 340-349.

Beydoun, M.A., Shaked, D., Tajuddin, S.M., Weiss, J., Evans, M.K., Zonderman, A.B., 2020c. Accelerated epigenetic age and cognitive decline among urban-dwelling adults. Neurology 94 (6), e613-e625.

Beydoun, M.A., Tajuddin, S.M., Dore, G.A., Canas, J.A., Beydoun, H.A., Evans, M.K., Zonderman, A.B., 2017. Vitamin D Receptor and Megalin Gene Polymorphisms Are Associated with Longitudinal Cognitive Change among African-American Urban Adults. J Nutr 147 (6), 1048-1062.

Beydoun, M.A., Tajuddin, S.M., Shaked, D., Beydoun, H.A., Evans, M.K., Zonderman, A.B., 2019a. One-carbon metabolism gene polymorphisms are associated with cognitive trajectory among African-American adults. Neurobiol Aging 84, 238 e235-238 e218.

Beydoun, M.A., Weiss, J., Beydoun, H.A., Hossain, S., Maldonado, A.I., Shen, B., Evans, M.K., Zonderman, A.B., 2021b. Race, APOE genotypes, and cognitive decline among middle-aged urban adults. Alzheimers Res Ther 13 (1), 120.

Beydoun, M.A., Weiss, J., Obhi, H.K., Beydoun, H.A., Dore, G.A., Liang, H., Evans, M.K., Zonderman, A.B., 2019b. Cytokines are associated with longitudinal changes in cognitive performance among urban adults. Brain Behav Immun 80, 474-487.

Delis, D.C., Freeland, J., Kramer, J.H., Kaplan, E., 1988. Integrating clinical assessment with cognitive neuroscience: construct validation of the California Verbal Learning Test. J Consult Clin Psychol 56 (1), 123-130.

Ekstrom, R.B., French, J.W., Harman, H.H., Dermen, D., 1976. Manual for kit of factor referenced cognitive tests. Educational Testing Service, Princeton, NJ.

Fanelli Kuczmarski, M., Crawford, S.B., Sebastian, R.S., Beydoun, M.A., Goldman, J.D., Moshfegh, A.J., Evans, M.K., Zonderman, A.B., 2024. Association between Flavonoid Intake and Cognitive Executive Function among African American and White Adults in the Healthy Aging in Neighborhoods of Diversity across the Life Span (HANDLS) Study. Nutrients 16 (9).

Folstein, M.F., Folstein, S.E., McHugh, P.R., 1975. “Mini-mental state”: a practical method for grading the cognitive state of patients for the clinician. Journal of psychiatric research 12 (3), 189-198.

Hossain, S., Beydoun, M.A., Kuczmarski, M.F., Tajuddin, S., Evans, M.K., Zonderman, A.B., 2019. The Interplay of Diet Quality and Alzheimer's Disease Genetic Risk Score in Relation to Cognitive Performance Among Urban African Americans. Nutrients 11 (9).

Hossain, S., Beydoun, M.A., Weiss, J., Kuczmarski, M.F., Evans, M.K., Zonderman, A.B., 2020. Longitudinal associations between dietary quality and Alzheimer's disease genetic risk on cognitive performance among African American adults. Br J Nutr 124 (12), 1264-1276.

Morris, J.C., Heyman, A., Mohs, R.C., Hughes, J.P., van Belle, G., Fillenbaum, G., Mellits, E.D., Clark, C., 1989. The Consortium to Establish a Registry for Alzheimer's Disease (CERAD). Part I. Clinical and neuropsychological assessment of Alzheimer's disease. Neurology 39 (9), 1159-1165.

Morris, J.C., Mohs, R.C., Rogers, H., Fillenbaum, G., Heyman, A., 1988. Consortium to establish a registry for Alzheimer's disease (CERAD) clinical and neuropsychological assessment of Alzheimer's disease. Psychopharmacol Bull 24 (4), 641-652.

Nguyen, H.T., Kitner-Triolo, M., Evans, M.K., Zonderman, A.B., 2004. Factorial invariance of the CES-D in low socioeconomic status African Americans compared with a nationally representative sample. Psychiatry Res 126 (2), 177-187.

Reitan, R., 1992. Trail Making Test: Manual for Administration and Scoring. Reitan Neuropsychological Laboratory, Tucson, AZ.

Rouleau, I., Salmon, D.P., Butters, N., Kennedy, C., McGuire, K., 1992. QUANTITATIVE AND QUALITATIVE ANALYSES OF CLOCK DRAWINGS IN ALZHEIMERS AND HUNTINGTONS-DISEASE. Brain and Cognition 18 (1), 70-87.

Schretlen, D., Bobholz, J.H., Brandt, J., 1996. Development and psychometric properties of the Brief Test of Attention. Clinical Neuropsychologist 10 (1), 80-89.

Wechsler, D., 1981. WAIS-R manual. The Psychological Corporation, Cleveland.

Wilkinson, G.S., 1993. Wide Range Achievement Test–Revision 3. Jastak Association, Wilmington, DE.
